# Double-Blind Acupuncture Needle: A Potential Tool to Investigate the Nature of Pain and Pleasure

**DOI:** 10.1155/2013/825751

**Published:** 2013-08-18

**Authors:** Nobuari Takakura, Miho Takayama, Akiko Kawase, Ted J. Kaptchuk, Hiroyoshi Yajima

**Affiliations:** ^1^Department of Acupuncture and Moxibustion, Faculty of Health Sciences, Tokyo Ariake University of Medical and Health Sciences, 2-9-1 Ariake, Koto-ku, Tokyo 135-0063, Japan; ^2^Department of Physiology, Showa University School of Medicine, Tokyo 142-8555, Japan; ^3^Japan School of Acupuncture, Moxibustion and Physiotherapy, Tokyo 150-0031, Japan; ^4^Program in Placebo Studies, Beth Israel Deaconess Medical Center, Harvard Medical School, Boston, MA 02215, USA

## Abstract

*Background*. Most of our knowledge about similarities in the neural processing of painful and pleasant sensations in the brain derives from studying each phenomenon separately. Patients often feel pain induced by acupuncture, which is noxious stimulation having the symbolic message of the cure, as pleasant. *Objectives*. We investigated whether the double-blind acupuncture needles are a potential tool to investigate coinciding pain and pleasant events. *Methods*. Participants were 109 healthy acupuncture students. An acupuncturist applied the double-blind placebo and the matching penetrating needle at bilateral forearm of each subject, one needle on each side of the arm. We asked the subjects to rate the pain associated with needle application and its unpleasantness or pleasantness on a visual analogue scale. *Results*. Of 65 penetrating needle applications that elicited pain, 29 (45%) subjects did not describe the pain as being unpleasant, and interestingly, 18 (28%) subjects described the needle insertion as pleasant. There was no significant difference in reported pain intensity between penetrating needles elicited pain that elicited a pleasant sensation and those that elicited an unpleasant sensation (P = 0.34). *Conclusions*. The double-blind acupuncture needles can be a potential tool for investigating the concomitant hedonic (pleasure) experience of pain.

## 1. Introduction

Pain and pleasure have been considered opposites [1]. Most of our knowledge regarding pain and pleasure is obtained from independent studies of each phenomenon [1]. Needle insertion during acupuncture treatment which is one of the most popular complementary and alternative medicines is a noxious stimulation, but at the same time it gives patients an expectation of beneficial cure [2]. Very interestingly, patients who receive acupuncture treatment sometimes say that they feel pleasant having pain induced with acupuncture despite its invasive nature. The cause of this paradoxical phenomenon might be the effect of symbolic message of cure which had impact on the subjective utility of pain [1]. To the best of our knowledge, however, there has been no scientific study to show the relationship between pain associated with acupuncture needle insertion and its affective state under double-blind conditions. If it is scientifically confirmed that the penetration of an acupuncture needle can elicit both pain and pleasure concurrently, it can serve as a tool for investigating the neural mechanism for aversive stimuli that may have the subjective utility, which might close the gap between the pain and pleasure research field.

 The aim of this study is to find out whether the double-blind acupuncture needles [3, 4] are latent tool to investigate coinciding pain and pleasure events.

## 2. Methods

### 2.1. Participants

Participants were 109 healthy volunteers (mean ± SD age of 28.6 ± 7.5 years; 64 males and 45 females) who were acupuncture students from the Japan School of Acupuncture, Moxibustion and Physiotherapy, Tokyo, Japan. We recruited a well-educated and experienced acupuncturist. Prior to the study, its purpose and format were explained to the participants, all of whom provided written consent. The study was approved by the Ethics Committee of Showa University School of Medicine.

### 2.2. Acupuncture Needles

**Figure 1:**
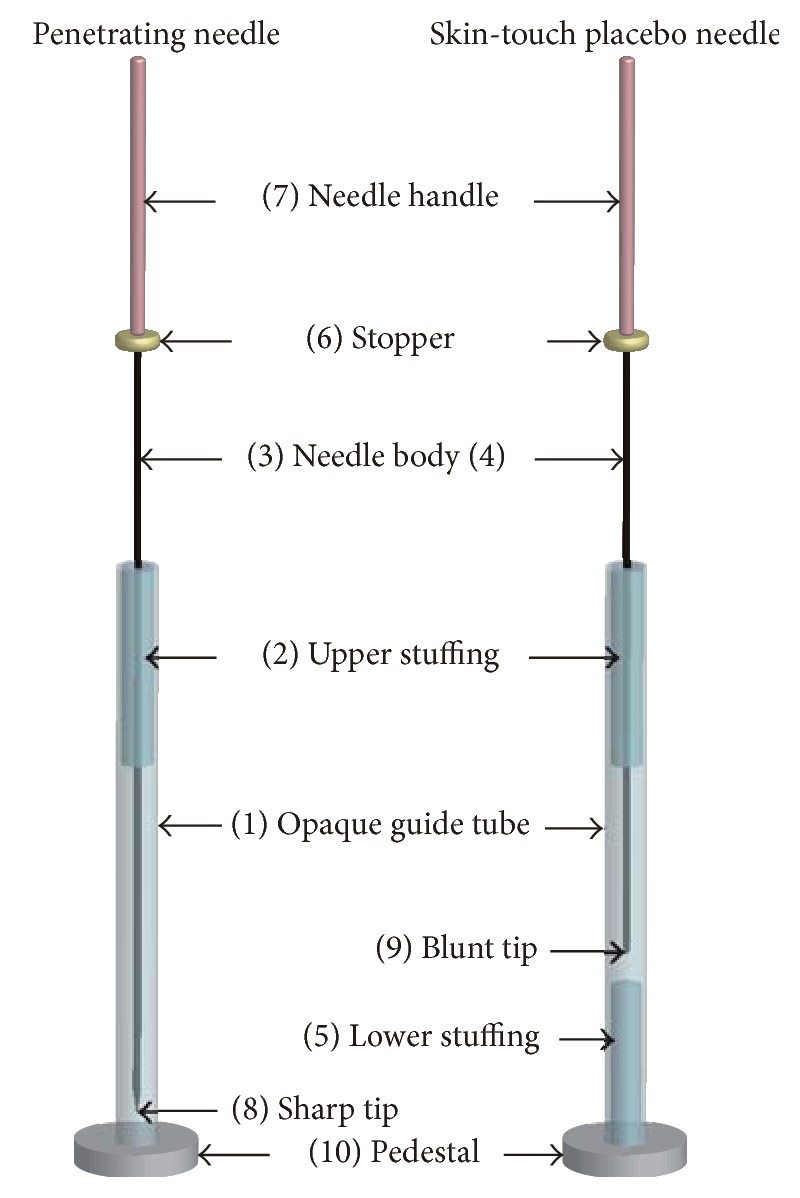
The design of the double-blind (practitioner-patient blinding) skin-touch placebo needle and matching penetrating needle [3, 4]. Both needles comprise an opaque guide tube (1) and upper stuffing (2). The body of the penetrating needle (3) is longer than the guide tube by an amount equal to the insertion depth, but the needle body of the skin-touch placebo needle (4) is just long enough to allow its blunt tip to press against the skin when the needle body is advanced as far as possible. The skin-touch placebo needle contains lower stuffing (5). Both needles have a stopper (6), which prevents the needle handle (7) from advancing further when the sharp tip of the penetrating needle (8) or the blunt tip of the skin-touch placebo needle (9) reaches the specified position. The pedestal (10) on each needle is adhesive, allowing it to stick firmly to the skin surface.

We used two types of needles in this study (Figure 1) [3, 4]: (1) the double-blind “skin-touch placebo needle,” the tip of which presses against the skin but cannot penetrate it and (2) the matching real “penetrating needle.” These needles were developed for research employing Japanese style of acupuncture. For both patients and practitioners, the appearance and feel of the skin-touch placebo and penetrating needles are virtually identical and practically indistinguishable. These needles have been described in detail elsewhere [3, 4]. For both needles, the upper stuffing gives resistance to the needle body during its passage through the guide tube. The blunt tip of the skin-touch placebo needle meets some resistance from the lower stuffing in the guide tube to give the impression that it penetrates. Thus practitioners cannot easily distinguish penetration or needle touching the skin, and they remain blind to application as to whether the needle application is real or placebo. For patient blinding, the skin-touch placebo needles have been well validated. Even for the penetrating needles, the patients misidentify a certain amount of them as skin-touch needle [4]. Further, the patients are uncertain of their judgments for majority of the correctly identified needles [4].

### 2.3. Protocol

To induce pain under double-blind conditions, we employed 109 double-blind (practitioner-patient blinding) skin-touch placebo needles with a blunt tip and 109 identical-looking penetrating needles with 10 mm insertion depth and 0.16 mm in diameter (Figure 1). Before the trial, the 109 subjects and the practitioner (Japanese licensed acupuncturist; woman; 39 years old; acupuncture experience, 7 years) were informed of the potential use of skin-touch placebo needles.

 Each needle was sealed in a small opaque container. The containers were sorted into pairs—one with a penetrating needle, the other with a skin-touch placebo needle—and each pair of containers was sealed in an opaque envelope and sterilized. We prepared one envelope which contained the two opaque containers per each subject. The acupuncturist blindly selected a needle container from the envelope and then applied the needle. Next, the acupuncturist applied the other needle to the subject. In this way, penetrating and skin-touch placebo needle were randomly assigned. The two insertion points for each subject were the bilateral Triple Energizer-5 (TE5) acupoints on the posterior surface of the forearm [5], one needle on each side of the arm. Each needle was applied using the alternating twirling technique (rotating the needle clockwise and counterclockwise alternately) until the stopper made contact with the top of the guide tube [3, 4]. Immediately after, the practitioner pulled the needle out to the initial position. There was no retention time.

 After each needle application, we asked the subject to rate the penetration/penetration-like pain on a visual analogue scale (VAS) ranging from 0 (no pain) to 100 (the most severe pain imaginable) [4]. We also asked each subject to rate the pleasantness or unpleasantness associated with each needle sensation on a VAS ranging from −100 (extremely unpleasant) to 0 (neither unpleasant nor pleasant) to +100 (extremely pleasant). In order to evaluate success of blinding, each subject and the practitioner were asked to guess whether a needle was “penetrating” or “skin touch” after each needle removal [3, 4]. They were also asked to report their confidence on a VAS, ranging from 0% (no confidence) to 100% (complete confidence), in identification of needle authenticity [3, 4].

### 2.4. Statistical Analysis

We performed statistical comparisons for VAS scores in penetration/penetration-like pain for penetrating needle with pleasant sensation versus penetrating needle with unpleasant sensation by Mann-Whitney *U* test, because the VAS score obtained in this study was revealed not to be normally distributed (*P* < 0.01) by Shapiro-Wilk test. All statistical analyses were performed using IBM SPSS Statistics 18 (SPSS Japan Inc., an IBM company).

## 3. Results

### 3.1. Needle Pain and Pleasure

**Figure 2:**
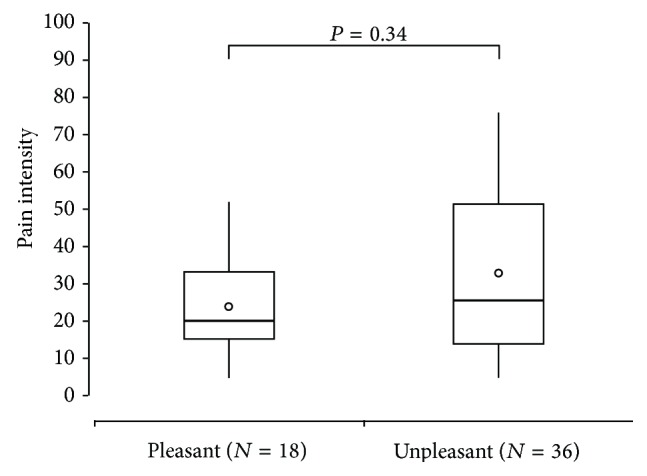
Pain intensity for the penetrating needles with a pleasant sensation and with an unpleasant sensation. There was no significant difference between the penetrating needles with the pleasant sensation and those with the unpleasant sensation in the subjects who had pain associated with needle insertion. Pain intensity was expressed on 100 mm visual analogue scale. The top, middle, and bottom lines of the boxes correspond to the 75th, 50th, and 25th percentiles, respectively. The whiskers extend from 10th to the 90th percentile. The circles indicate the arithmetic mean.

Of 109 penetrating needles, 65 (59.6%) needles elicited needle penetration pain (pain intensity: median (mean ± SD), 21.0 (29.8 ± 22.8) on the VAS). Of the 65 penetrating needles, 29 (44.6%) subjects did not describe the pain as unpleasant, and interestingly, 18 (27.7%) subjects described the needle insertion as a pleasant experience; the rest of 11 subjects described it as neither unpleasant nor pleasant. With respect to pain, there was no significant difference between the 18 penetrating needle applications that elicited a pleasant sensation (pain intensity: median (mean ± SD), 20.0 (24.0 ± 14.6)) and the 36 penetrating needle applications that elicited an unpleasant sensation (25.5 (33.8 ± 24.8)) (*P* = 0.34) (Figure 2). For the 11 subjects who described neither unpleasant nor pleasant sensation, pain intensity was 19.0 (26.4 ± 26.0).

### 3.2. Blinding

For practitioner blinding, 58 (53.2%) of 109 penetrating needles were correctly identified with 41.5 (48.7 ± 28.7) (median (mean ± SD)) % confidence on the VAS, and 51 (46.8%) were incorrectly identified with 0.0 (11.7 ± 21.2) % confidence. Of 109 skin-touch needles, 48 (44.0%) were correctly identified with 43.0 (44.8 ± 23.7) % confidence, and 61 (56.0%) were incorrectly identified with 0.0 (14.9 ± 25.6) % confidence.

For patient blinding, 24 (22.0%) of 109 penetrating needles were incorrectly identified with 70.0 (54.2 ± 39.6) % confidence, and the rest of the penetrating needles were correctly identified with 90.0 (85.6 ± 16.6) % confidence. Of 109 skin-touch placebo needles, 65 (59.6%) were correctly identified with 80.0 (76.2 ± 18.9) % confidence, and 44 (40.4%) were incorrectly identified with 80.0 (68.1 ± 37.4) % confidence.

## 4. Discussion and Conclusions

A certain amount of the healthy subjects who knew acupuncture is a therapeutic tool felt pain elicited with acupuncture needle insertion *per se* as pleasant. The acupuncture needle, which is noxious stimulation having the symbolic message of the cure, can be a potential tool for investigating the concomitant hedonic (pleasure) experience of pain under double-blind conditions.

Noxious stimulation that elicits pain having no meaning to the sufferer [1]—for example, being pricked by a splinter—almost always causes an unpleasant feeling. The subjects in this study had believed that acupuncture needle insertion is the beneficial cure. Therefore, the pain with acupuncture could have meaning—or the subjective utility—to a certain amount of the subjects. The results in this study suggest that suffering can be rewarding if it has meaning to the sufferer; pain-pleasure dilemmas in which a large reward is gained at the price of the small pain may be resolved not only through antinociceptive effects [1] but also by reversing the subjective emotional feeling of pain from unpleasant to pleasant. In healthy individuals who might have some reward expectation in this study as well as in extremely morbid situations or in the context of drug-addicted individuals [6], the painful event was felt to be pleasurable, which suggests that there is extensive overlap in the neural circuitry and chemistry of pain and pleasure processing at the system level [1] in healthy individuals. These blinding acupuncture needles have potential to obtain how the major regions involved in pain and pleasure processing, such as the nucleus accumbens, the pallidum, and the amygdala in the brain [1], are activated concomitantly under double-blind conditions using brain imaging techniques.

It is a limitation that the participants of this study might have positive impression on acupuncture treatment; therefore, we could not exclude the possibility that this might affect the results of this study. If the healthy subjects who do not believe in the benefit of acupuncture participated in this study, the results could clearly show the relationship between pain and positive reward expectation. Although the outcomes of this study could not be influenced by practitioner-oriented biases, patient-oriented biases might not be completely eliminated because the confidence scores were relatively high, which is another limitation of this study. However, using the skin-touch placebo needles which were well blinded to the subjects, we believe patient-oriented biases were minimal under the inherent difficulty in patient blinding from penetrating needles.

In conclusion, the double-blind acupuncture needles can be a potential tool for investigating the concomitant hedonic (pleasure) experience of pain.

## References

[1] Leknes S., Tracey I. (2008). A common neurobiology for pain and pleasure. *Nature Reviews Neuroscience*.

[2] Singh S., Ernst E. (2008). *Trick Or Treatment? Alternative Medicine on Trial*.

[3] Takakura N., Takayama M., Kawase A., Kaptchuk T. J., Yajima H. (2010). Double blinding with a new placebo needle: a further validation study. *Acupuncture in Medicine*.

[4] Takakura N., Takayama M., Kawase A., Yajima H. (2011). Double blinding with a new placebo needle: a validation study on participant blinding. *Acupuncture in Medicine*.

[5] World Health Organization (2008). *WHO Standard Acupuncture Point Locations in the Western Pacific Region*.

[6] Hampton T. (2006). A world of pain: scientists explore factors controlling pain perception. *Journal of the American Medical Association*.

